# Secret Remedies and Branded Products—III

**Published:** 1908-02-29

**Authors:** 


### Therapeutics.

### SECRET REMEDIES AND BRANDED PRODUCTS—III.

[It is the object of these articles to show how departure from rational prescribing has fostered and encouraged a system which is open to many grievous abuses, of which self-medication is not the least. The writer appreciates the fact that many valuable preparations are sold under proprietary and protected names, and wishes it clearly to be understood that products which are recognised by medical practitioners on account of their real utility are in no way called in question.]

In a previous article some indication was given as to the possible means provided by the existing law for the suppression of many forms of quackery. That these means are either impracticable or useless is clearly evidenced by the freedom permitted to the quacks in the continuance of their charlatanry, and special restrictive legislation is therefore an urgent necessity. In every European country, except our own, legislation of some kind restricts the sale of nostrums. In Austria, the sale of quack remedies is forbidden; new preparations may not be sold until their therapeutical action has been chemically tested and approved of by the medical authorities. Branded pharmaceutical specialities may be made only by registered pharmacists, and only drugs or chemicals admitted in medicine may enter into their composition; such products must possess some element of novelty or improvement in their physical properties, and the exact formulæ must be disclosed. The price of the preparation is regulated by the State, and in arranging this price no allowance is made for advertising expenses. Medicines whose composition has been intentionally misdescribed may not be sold even at the request of a medical practitioner.

In Germany, until recently, different States had different laws concerning secret remedies, and in some of the States where their sale was permitted, the police were accustomed to warn the public by official announcements in the newspapers that certain advertised remedies should be shunned, at the same time stating the composition and the cost of production. The advertisement of secret remedies was prohibited in Brunswick, Westphalia, Saxony. Altenburg and Wurtemburg, even when the formula was published. In Bavaria remedies could not be sold retail by chemists unless the composition was known to them, the constituent parts being drugs which could be legally retailed, and so long as the price of the remedy was no higher than was warranted by the price of its component parts. In Hesse and Hamburg, the sale of secret remedies was forbidden, but they could be supplied in exceptional cases on the prescription of a medical practitioner which had to be rewritten every time the remedy was sold. A new law, however, has just come into force in Germany, which practically puts an end to the sale and advertisement of all secret remedies. In France, the sale of medicines of which the composition is not disclosed, is prohibited, and, in fact, nowhere in Europe is the quack allowed a tenth part of the freedom permitted in this country.

The reason why charlatanry, as manifested in secret cures, has not been suppressed in this country is difficult to ascertain. Some have suggested as a reason that the Government is not anxious to deal with the matter, because the annual revenue is increased by this sale to the extent of over £300,000, but this suggestion may be dismissed as absurd. The cause is more probably due to the fact that our legislators, like newspaper proprietors, and the general public, cannot be made to believe that the great bulk of secret medicines constitute such a grave danger to the public health and such an abominable fraud upon themselves. Publishers of newspapers have often been accused of knowingly perpetrating a gross offence by admitting to their columns quack medicine advertisements. This accusation is unjust and the admission of such announcements is in the main due to ignorance; and unfortunately, the ignorance of the offending publisher is sometimes fostered by the knowledge that his own medical adviser prescribes for him some secret compound or proprietary product which he can obtain from any chemist in the original package without a prescription.

It is not necessary in these columns to give reasons for classifying the vast majority of advertised nostrums as frauds, but as the extent to which makers of fraudulent nostrums will go when occasion arises may not be properly realised, the following facts ought to be placed on record:

Until a High Court interpretation of the Pharmacy Act, 1868, made it clear that proprietary medicines containing morphine, strychnine and all other scheduled poisons must bear a label on which is printed the name of the poison and the word "poison," a large number of poisonous preparations which the makers represented to be harmless, were sold to the public without restriction. At that time there was a preparation on the market held out as a certain cure for all affections of the lungs, the sole medicinal ingredient of which was morphine. When the law, as interpreted by the High Court, was enforced, the makers of the preparation had to decide whether they would label it poison or leave out the sole medicinal ingredient; they chose the latter alternative, and yet to-day the "cure for lung complaints," as a result of extensive advertising, has a phenomenal sale.

A "tonic," which previous to the High Court decision, owed its properties to strychnine, is now sold—with precisely the same recommendations—without the strychnine. In nearly every other country but our own, acetanilide is treated as a scheduled poison, but a widely advertised proprietary headache "cure," to which several deaths are directly traceable, composed of this product, is freely sold in England. Now, four years ago, a new interpretation was placed by the Court on the Medicine Stamp Act, and one result is—it is unnecessary to go into the intricacies of this Act here—that this penny headache powder, if sold by a dealer who does not hold a patent medicine license, has to bear a 1½d. stamp, although it is free of duty when sold by licensed dealers. Here was a difficulty which might have perplexed an ordinary trader, but not so the proprietors of this headache " cure " ; they simply evaded the Act by substituting phenacetin for acetanilide, making a slight technical alteration on the wrapper, which is not noticed by the ordinary purchaser, the result being that a person who buys this proprietary article from a licensed dealer has a totally different powder from that purchased from an unlicensed dealer. The knowledge that such evil practices exist creates a feeling of revolt in honest persons; and yet similar methods are adopted by manufacturers who insult the physician's intelligence by telling him how to prescribe.

When occasion requires, these manufacturers are just as nimble as the proprietors of nostrums advertised to the public. Thus it is provided by Section 8 of the National Food and Drugs Act, which came into force in America at the beginning of 1906, that preparations containing acetanilide, or any of its derivatives, must bear a label stating the quantity of such ingredients; the effect of the enforcement of this Act has been to cause makers of certain largely prescribed antipyretics to alter the compositions of these preparations very materially. For instance, one well-known article, which formerly was said to contain acetanilide, sodium bicarbonate and caffeine, is now described as a mixture containing 350 grains of acetphenetidine per ounce. Other instances of a more striking character may be cited in due course, but in the meantime this one example suffices to illustrate how accommodating proprietors of fancy named articles can be when necessary. In this country no obligation is placed upon manufacturers of branded products to disclose the composition of such preparations; it is true that many owners of proprietary articles make some sort of statement to medical practitioners, but it is equally true that these statements are very often utterly misleading.

